# Trends in survival from myeloma, 1990–2015: a competing risks analysis

**DOI:** 10.1186/s12885-021-08544-7

**Published:** 2021-07-16

**Authors:** Mary Jane Sneyd, Andrew R. Gray, Ian M. Morison

**Affiliations:** 1 Hugh Adam Cancer Epidemiology Unit, Department of Preventive and Social Medicine, University of Otago, PO Box 56, Dunedin, 9054 New Zealand; 2 Biostatistics Centre, University of Otago, Dunedin, New Zealand; 3 Department of Pathology, University of Otago, Dunedin, New Zealand

**Keywords:** Myeloma, Survival, Competing risk regression

## Abstract

**Background:**

Myeloma survival has greatly increased over past decades. We investigated trends in survival over time in New Zealand by age, ethnicity, and geography and thus examined potential inequalities among these population subgroups.

**Methods:**

From data supplied by the New Zealand Ministry of Health, all new diagnoses of multiple myeloma (ICD-10 code C90) between 1990 and 2016 were extracted, as well as their matched mortality data. Cox’s proportional hazards regression and competing risks regression were used to estimate multivariable survival functions.

**Results:**

Between 1 January 1990 and 1 December 2015, 6642 myeloma cases were registered by the New Zealand Cancer Registry. Although survival from myeloma increased substantially from 1990–1994 to 2010–2015, 5-year survival was still only about 60% in 2010–2015. The greatest improvement in survival was for people aged 60–69 years at diagnosis. Using Cox’s proportional hazards regression, Māori showed an increased risk of myeloma death but this was predominantly due to differences in competing risks among ethnic groups. Competing risks analysis found the greatest improvement in myeloma survival in Pacific Islanders, and in 2010–2015 Māori had better survival than other ethnicities. Myeloma survival improved significantly over time in all regional health authorities but in all time periods the Central and Southern regions had significantly poorer survival than the Midland region.

**Conclusions:**

Improvements in myeloma survival have been unequal across subgroups and regions in New Zealand. Detailed information about utilization of chemotherapeutic agents and transplantation in New Zealand is not available. This information, as well as more detailed hematological data, is essential to further explore the relationships and reasons for differing myeloma survival in population subgroups of New Zealand.

**Supplementary Information:**

The online version contains supplementary material available at 10.1186/s12885-021-08544-7.

## Background

Multiple myeloma (plasma cell myeloma) is a hematological cancer involving the proliferation of plasma cells within the bone marrow. It primarily affects older adults. For unknown reasons incidence rates are consistently higher in men than women, and higher in black populations worldwide [1, 2] and in New Zealand Māori [3].

Although myeloma remains incurable, survival has greatly increased over the past few decades, particularly in younger patients, due to stem cell transplantation and the increasing availability of drugs such as thalidomide, lenalidomide and bortezomib [4]. However, probably due to the expense of and access to these therapies, there is some evidence that survival has not increased uniformly across subgroups defined by socioeconomic status [4, 5] and ethnicity [6]. Although there may be some differences in the biology of myeloma in different ethnicities [7], blacks and whites receiving the same transplantation treatment for myeloma had very similar outcomes [8, 9], suggesting that differences in access to treatment are likely to be of greater importance in eventual outcomes.

Stem cell transplantation has been widely available for over 20 years in New Zealand. In 1996 the first study of high-dose chemotherapy combined with autologous stem cell transplantation that showed improved outcomes for myeloma patients was published [10]. Since then, this procedure has become the standard of care for over two decades [11]. Conditioning chemotherapy before transplantation has been predominantly melphalan in New Zealand and more recently some selected patients will have received tandem transplantation.

Bortezomib (an anti-cancer proteasome inhibitor drug) was funded by Pharmac (the Pharmaceutical Management Agency responsible for deciding which pharmaceutical products are subsidized for use in New Zealand) from May 2011 for patients with newly diagnosed myeloma or those with relapse and no previous exposure to bortezomib. Prior to this date it was only available to a subset of patients with a poor prognosis - those with translocation t(4;14) myeloma - under the Named Patient Pharmaceutical Assessment (NPPA) mechanism.

We aimed to investigate the change in survival from myeloma over time in New Zealand by age, ethnic group and geography and thus examine potential inequalities among these population groups.

## Materials and methods

In New Zealand, all cancer diagnoses made since July 1994, except non-melanoma skin cancer, are notified to the New Zealand Cancer Registry (NZCR) under the Cancer Registry Act 1993. Prior to 1994, cancers were notified to the NZCR but with variable completeness: cancers reliant on the public hospital system for diagnosis (for example, myeloma) were notified whereas those diagnosed in general practice (for example, melanoma) or in private hospitals were less likely to be notified. From data supplied by the Statistical Services of the Ministry of Health, all new diagnoses of multiple myeloma or plasmacytoma (ICD-10 code C90) registered with the NZCR between 1990 and 2016 were extracted. ICD-10 code C90 is hereafter referred to as myeloma for simplicity. Over the time period covered by this study, the NZCR changed from ICD-9 to ICD-10 cancer codes. Some retrospective conversions from ICD-9 to ICD-10 for myeloma and plasmacytoma were found to be inaccurate so codes prior to 2000, when ICD-10 was introduced, were corrected as necessary. Diagnoses listed as ‘suspicious for’ or ‘probable’ myeloma or plasmacytoma were not included.

Mortality data were obtained from the mortality collection of the Ministry of Health [12]. This information was available for 1988 to end 2015 and included both ICD-9 and ICD-10 codes so no retrospective conversions were required. These data were matched to the Cancer Registry data using national health index (NHI) numbers (the patient identifier used in the New Zealand health system) to obtain information about deaths in people with a myeloma diagnosis. The main cause of death was categorized as either myeloma or any other cause. Because of some incomplete mortality data prior to 1990, analyses included people diagnosed between 1 January 1990 and 1 December 2015 (the most recent year of mortality data available). Patients still alive were censored on 1 December 2015. Examination of the mortality data revealed that it was extremely likely that some patients were missing their date of death and these patients were therefore excluded from the analysis. There were 43 such patients, comprising: patients aged over 100 years at the censor date; patients aged 90–99 years at the censoring date and who had survived 5+ years with myeloma; and patients aged 85–89 years at the censoring date and who had survived 8+ years with myeloma [13].

Data for sex, date of diagnosis, date of birth, residential location, and ethnicity, were used as recorded by the NZCR or NHI databases of the Ministry of Health. Ethnic classifications used prioritized ethnicity groups as defined by Statistics New Zealand whereby each individual is allocated to a single ethnicity based on the following priority: Māori, Pacific peoples, Asian, other groups except New Zealand European, and finally New Zealand European. Ethnicity was ultimately categorized into three groups: Māori, Pacific Islanders (PI), and non-Māori non-Pacific Islanders (NMNPI). Residential location was defined as the District Health Board area in which the person lived at the time of diagnosis. These 21 areas were grouped in the standard manner into the four regional health authorities (RHA): Northern RHA (Northland, Waitemata, Auckland, Counties Manukau), Midland RHA (Waikato, Lakes, Bay of Plenty, Tairawhiti, Taranaki), Central RHA (Hawkes Bay, MidCentral, Whanganui, Hutt, Capital & Coast, Wairarapa, Nelson Marlborough) and Southern RHA (West Coast, Canterbury, South Canterbury, Southern).

For a simple visual display of trends in myeloma survival, crude myeloma-specific survival, stratified by year or age of diagnosis categories and ethnicity, was displayed using the Kaplan-Meier method, with year of diagnosis categorized into 5-year groups from 1990 to 2009 (i.e., 1990–1994, 1995–1999, etc.) and 6 years for 2010–2015.

There are two commonly-used methods to analyse multivariable survival data: Cox’s proportional hazards regression and competing risks regression using Fine and Gray’s proportional subhazards model. The outcome of interest determines which method is the appropriate one to use. Competing risks regression is the correct technique to use when investigating prognostic questions, as in this paper, rather than issues of etiology, in which case it is often preferable to use Cox’s proportional hazards regression. However, as a large proportion of the literature on survival after myeloma uses Cox’s proportional hazards regression, with other causes of death censored, rather than competing risks regression, Cox’s proportional hazards regression was used to estimate multivariable survival functions and allow comparison with other published work. As myeloma patients tend to be elderly and may have reasonably long survival time, deaths from other (non-myeloma) causes are common in these patients. In the presence of competing failure events (i.e. deaths from other causes) that prevent a death from myeloma occurring, a standard analysis using Cox’s proportional hazards regression produces survival curves that are inappropriate as they apply only for a hypothetical universe in which these competing events do not occur [14]. Censoring under Cox’s proportional hazards regression assumes that a person is still at risk of a myeloma death after the censoring date, even though people who had died of another cause could no longer die from myeloma [15].

For these reasons, the cumulative incidence of myeloma death with competing risks was calculated to address the aims listed earlier. Competing risks regression provides an appropriate model to produce incidence curves that represent the observed data when the outcome of interest is prognosis. Patients who died from causes other than myeloma were included as competing events (deaths) and therefore no longer at risk of developing myeloma, instead of being censored on their date of death (but still considered at risk of developing myeloma) as occurs with Cox’s proportional hazards regression for cause-specific survival.

Competing risks regression was carried out according to the method of Fine and Gray [16]. Briefly, this method calculates the cumulative incidence of cause-specific death (in this case a death from myeloma) and reports subdistribution hazard ratios (SHR) associated with increased mortality while controlling for covariates and accounting for competing risks in the same model. The SHR can be simply interpreted as the adjusted risk of death compared to the reference population over the entire time period or within specified time periods*.* Because of non-linear effects of age at diagnosis and year of diagnosis, and their significant interaction, age at diagnosis was included as four categories (< 60 years, 60–69 years, 70–79 years, 80+ years) and year of diagnosis was included as five categories (1990–1994, 1995–1999, 2000–2004, 2005–2009, 2010–2015) in the regression models. *P*-values for pairwise comparisons used the lincom command rather than the margins command in Stata.

All analyses were performed in Stata 15. Two-sided 95% confidence intervals (CI) are presented and, in all analyses, 2-sided *p*-values ≤0.05 were considered statistically significant.

## Results

In New Zealand, between 1 January 1990 and 1 December 2015, 6642 diagnoses of myeloma were reported to the NZCR (Table 1). Over half of these were men (56.8%), and 53.4% of diagnoses occurred in people aged 70 years and over, with median ages at diagnosis of 72 years in NMNPI, 64 years in Māori, and 66 years in PI.

**Table 1:** Patient characteristics

		Non-Māori non-Pacific Islander (NMNPI)	Māori	Pacific Islander (PI)	Overall
	*n* = 6642	n (%)	n (%)	n (%)	n (%)
Sex	Male	3298 (57.6)	310 (52.2)	166 (51.1)	3774 (56.8)
	Female	2425 (42.4)	284 (47.8)	159 (48.9)	2868 (43.2)
Age in years	< 60	1089 (19.0)	233 (39.2)	92 (28.3)	1414 (21.3)
	60–69	1380 (24.1)	182 (30.6)	118 (36.3)	1680 (25.3)
	70–79	1799 (31.4)	132 (22.2)	80 (24.6)	2011 (30.3)
	80+	1455 (25.4)	47 (7.9)	35 (10.8)	1537 (23.1)
Median age and interquartile range (years)		72 (18)	64 (16)	66 (14)	71 (18)
Regional health authority region of residence	Northern	1702 (29.7)	210 (35.4)	230 (70.8)	2142 (32.3)
	Midland	1143 (20.0)	220 (37.0)	15 (4.6)	1378 (20.8)
	Central	1369 (23.9)	120 (20.2)	45 (13.9)	1534 (23.1)
	Southern	1478 (25.8)	44 (7.4)	27 (8.3)	1549 (23.3)
	missing	31 (0.5)	0	8 (2.5)	39 (0.6)

Approximately 4.9% of diagnoses were in PI and 8.9% were in Māori, compared to 2013 Census data [17] where about 7% of the total population was listed as PI and 15% were Māori.

After exclusion of the 19 people known to have died overseas and missing cause of death data, and the 43 with assumed missing dates of death, 6580 people diagnosed with myeloma remained for the survival analysis. Of these people, 3535 (53.7%) died of their disease before 1 December 2015, 1227 (18.6%) died from other causes and the remainder (*n* = 1818) was still alive on 1 December 2015.

Crude survival after a myeloma diagnosis is shown by the Kaplan-Meier curves in Additional file 1. Crude survival has improved considerably since 1990–1994 and worsens with increasing age at diagnosis. Crude survival appears better in PI than either Māori or NMNPI peoples.

To adjust for potential confounding factors, such as the age distribution of different ethnic groups, and to make allowance for death from non-myeloma causes, competing risks regression was performed. Table 2 shows the percentage of cases overall who have survived for various times after their myeloma diagnosis. Survival from myeloma has increased substantially in both sexes from 1990–1994 to 2010–2015 but 5-year survival still remains at about 60% in 2010–2015.

**Table 2:** Percentage of cases that have survived, by time since diagnosis and year of diagnosis, using competing risks regression

	Survival %							
	6 months		1 year		2 years		5 years	
Year of diagnosis	Male	Female	Male	Female	Male	Female	Male	Female
1990–1994	79.0	77.7	70.5	68.7	57.5	55.2	37.9	35.3
1995–1999	80.7	79.4	72.7	71.0	60.4	58.2	41.3	38.7
2000–2004	82.6	81.4	75.2	73.7	63.8	61.7	45.6	42.9
2005–2009	85.5	84.5	79.2	77.9	69.2	67.3	52.5	50.0
2010–2015	89.8	89.1	85.2	84.2	77.6	76.1	64.1	62.0

**Fig. 1:**
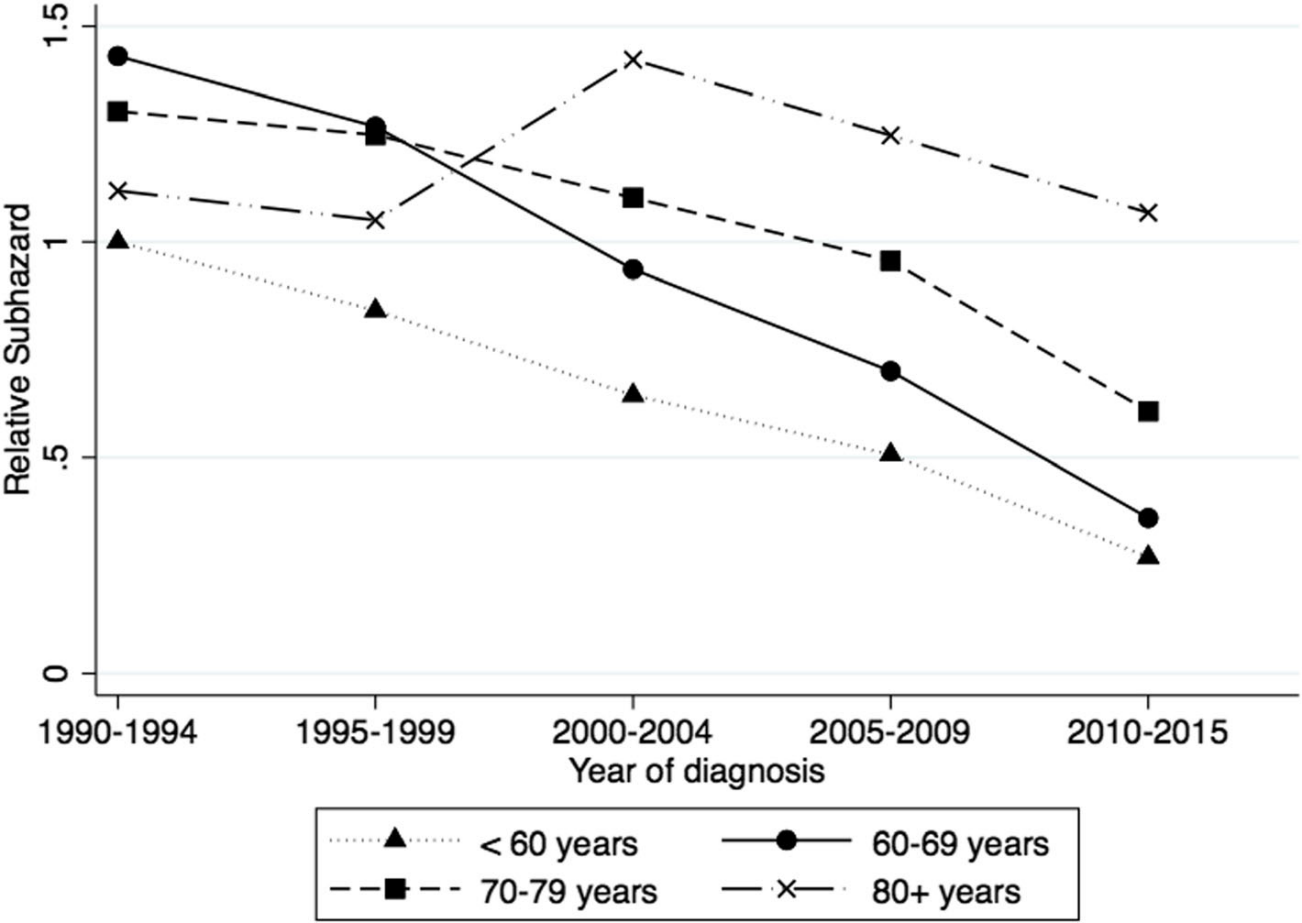
Adjusted subdistribution hazard ratio by year and age at diagnosis, using multivariable competing risks regression

Survival from myeloma varies considerably by age at diagnosis as well as year of diagnosis. In Fig. 1 the adjusted subdistribution hazard ratio (SHR) for dying from myeloma by year of and age at diagnosis can be seen, showing effect modification of the age association by diagnosis period (interaction *p* < 0.001). For the two youngest age groups (< 60 years and 60–69 years at diagnosis), the risk of dying from myeloma significantly decreased for all time periods from 2000–2004, compared to the risk in 1990–1994 (*p* < 0.001 for both age groups in all periods). For those aged 70–79 years, the risk significantly improved from 2005–2009 but for 80+ year old patients there was no overall improvement during the study period. The greatest improvement from 1990–1994 to 2010–2015 was for people aged 60–69 years at diagnosis and whose SHR decreased by 0.89 in absolute terms (*p* < 0.001).

In 1990–1994, people aged 60–69 years had significantly worse survival than people aged < 60 years (*p* = 0.004) but there was no significant difference in survival among the other age groups. By 2010–2015, people aged < 60 years and 60–69 years had the best survival of the age groups, with the two oldest age groups having significantly worse survival than other age groups. However, in 2010–2015, people aged 80+ years at diagnosis had significantly worse survival compared to all other age groups, including 70–79 years (*p* < 0.001 for all pairwise comparisons).

**Fig. 2:**
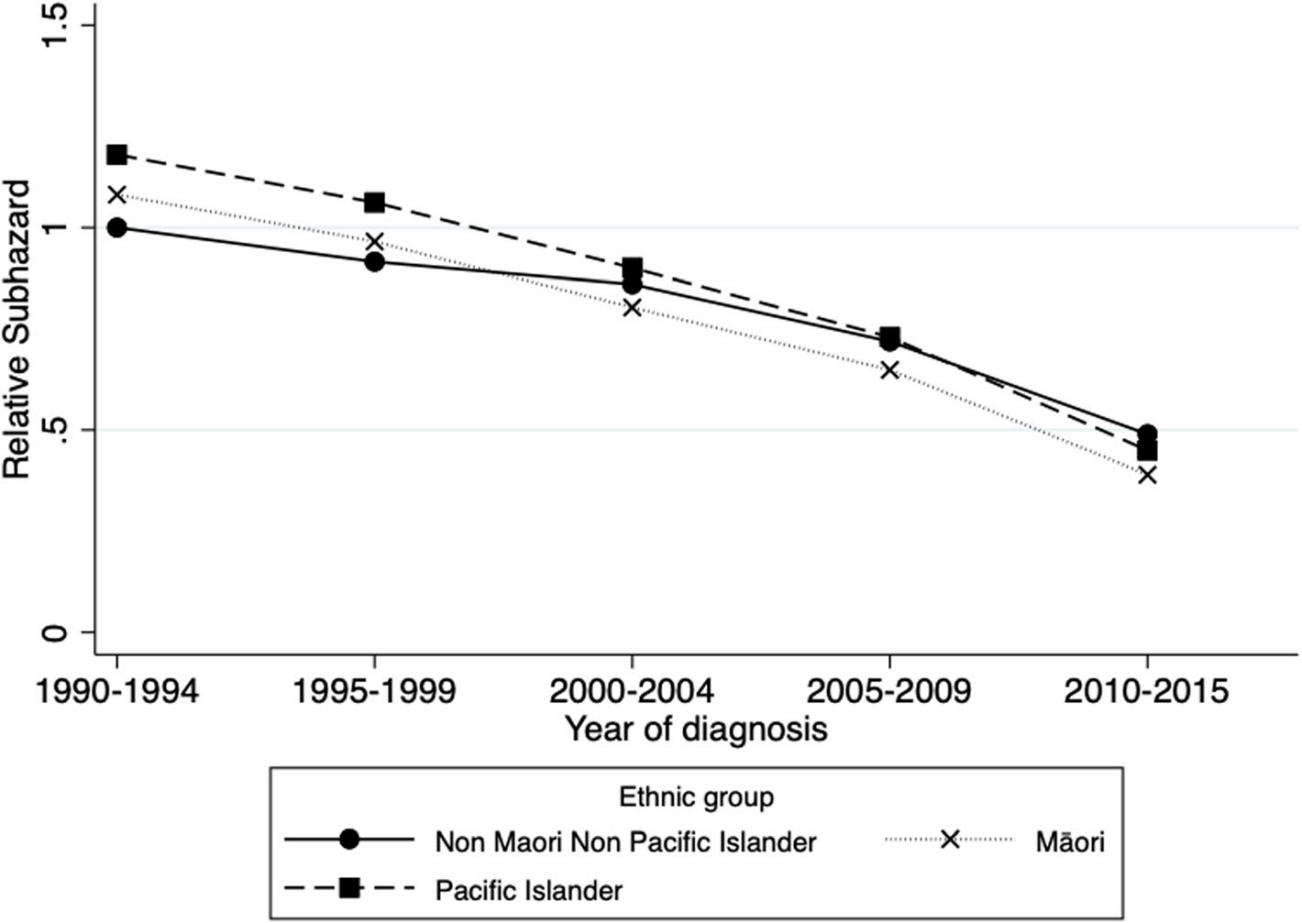
Adjusted subdistribution hazard ratio by diagnosis year and ethnic group using multivariable competing risks regression

Figure 2 shows the SHR by year of diagnosis and ethnic group. For NMNPI and PI, survival improved from 2000–2004 compared to 1990–1994, whereas in Māori all years improved on the previous ones. The survival of Māori and PI improved more from 1990–1994 to 2010–2015 than NMNPI peoples. There was no significant difference in survival between the ethnic groups in any years except in 2010–2015 when Māori had statistically significantly better survival than NMNPI (*p* = 0.002).

**Fig. 3:**
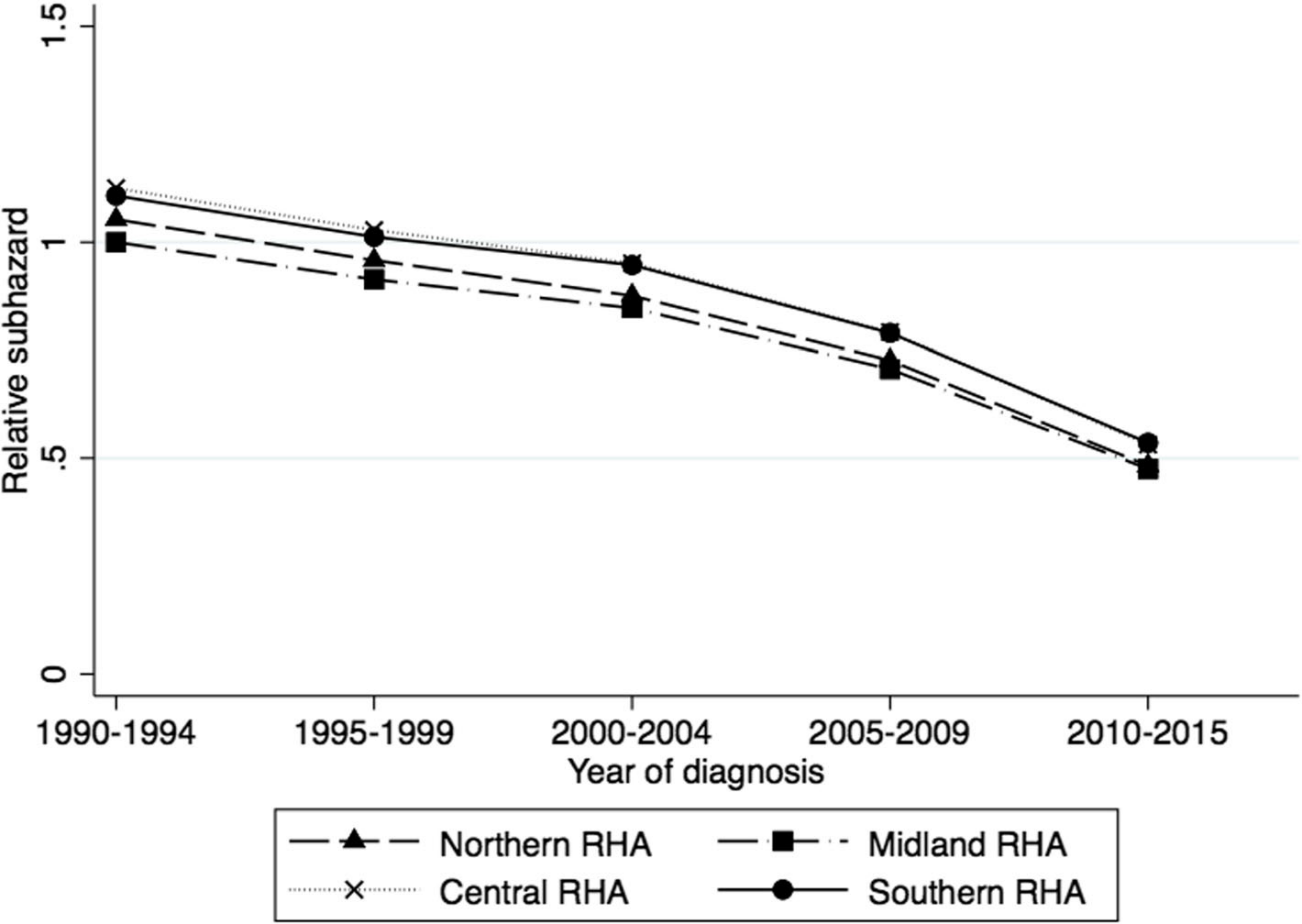
Adjusted subdistribution hazard ratio by diagnosis year and regional health authority (RHA), using multivariable competing risks regression

The survival from myeloma in all RHAs improved significantly over time (*p* < 0.001 for all RHAs) but the absolute improvement from 19901994 to 2010–2015 was greatest in the Central RHA, with a decrease in the fully adjusted SHR of 0.59 (Fig. 3). For all time periods examined, the Central and Southern RHAs had significantly poorer survival than the Midland RHA which had the best myeloma survival. However, in 2010–2005, survival in the Southern RHA was significantly worse than both the Northern and Midland RHAs (*p* = 0.036 and *p* = 0.022, respectively) and survival in the Central RHA was also poorer than the Northern and Midland RHAs (*p* = 0.053 and *p* = 0.034, respectively), albeit not statistically significantly so in the first case.

The results of multivariable Cox’s proportional hazards regression and competing risks regression are compared in Table 3 and Additional file 2. Because of the large table size required to show the age and year interaction effects, these have been included in Additional file 2. These analyses show that generally survival deteriorates with increasing age at diagnosis and that survival has improved with more recent diagnoses. Although it appeared (Additional file 2) with multivariable Cox’s proportional hazards regression that many age and year combinations had hazard ratios (HR) significantly different from the reference category (age < 60 years and diagnosed in 1990–1994), some of this was due to death from non-myeloma causes. Therefore, with competing risks regression, the point estimates of risk moved by varying amounts and directions. In particular the risks (SHR) for people aged 80+ years and diagnosed in all years except 2000–2004 were no longer significantly different from the reference group.

**Table 3:** Multivariable Cox’s proportional hazards regression and competing risk regression of myeloma survival

		Cox’s proportional hazards regression			Competing risk regression		
		Full model*			Full model*		
Variable		HR	*p* value	95% CI	SHR	p value	95% CI
Overall test for age at diagnosis and year of diagnosis interaction using the Wald test.**			< 0.001			< 0.001	
Ethnic group	non-Māori non-PI	1			1		
	Māori	1.32	< 0.001	1.17–1.49	1.12	0.076	0.99–1.28
	Pacific Islander	1.16	0.070	0.99–1.37	1.18	0.057	0.99–1.40
Sex	Male	1			1		
	Female	0.99	0.849	0.93–1.06	1.06	0.089	0.99–1.13
Regional Health Authority	Midland	1			1		
	Northern	1.04	0.452	0.94–1.14	1.04	0.381	0.95–1.15
	Central	1.19	0.001	1.07–1.31	1.13	0.022	1.02–1.25
	Southern	1.12	0.025	1.01–1.24	1.12	0.023	1.02–1.24

*HR* hazard ratio, *SHR* subdistribution hazard ratio

*Full model includes: year of diagnosis, age at diagnosis, ethnicity, regional health authority at diagnosis, sex, year of diagnosis and age at diagnosis interaction

** Data for individual categories of age at diagnosis and year of diagnosis are in Additional file 2

In the analyses by ethnic group, multivariable Cox’s proportional hazards regression showed that both Māori and PI had a significantly increased risk of dying from myeloma compared to NMNPI peoples, and it was considerably higher in Māori. However, when deaths from other causes were appropriately considered, Māori had no significant increase in risk whereas PI had an increase of 18% that was close to statistical significance (*p* = 0.057).

Consistent with the analysis of trends over time above, survival also varied significantly by RHA with both Cox’s proportional hazards regression and competing risks regression analysis (Table 3). After Cox’s proportional hazards regression, both the Central and Southern RHAs had significantly higher risk of death over the time period (*p* = 0.001 and *p* = 0.025, respectively) compared to the Midland RHA. Although the SHR for the Central RHA was reduced, these hazard ratios remained significantly above the Midland RHA after consideration of competing risks, showing that some of the difference between the Central and Midland RHAs, but not the other RHAs, was due to differences in competing risks.

There were no significant differences in survival after myeloma by sex over all age groups and years of diagnosis.

## Discussion

Despite making great strides in its treatment, multiple myeloma remains an incurable cancer. To the best of our knowledge, we have carried out the first competing risks analysis of trends in myeloma survival in New Zealand. For this study we used national, routinely collected and anonymized, population-based data of myeloma patients diagnosed between 1990 and 2015. Our analysis showed that survival has increased significantly for both men and women since 1990 but that these improvements were not consistent across age or ethnic groups. Using competing risks regression, we showed that although the point estimates of the SHR were slightly elevated in both Māori and Pacific people overall, these increases were not statistically significant, and since 2000–2004 the risk of myeloma death has been lower in Maori than in NMNPI. In contrast, there were significant differences in risk among the RHAs with Central and Southern RHAs having the highest risk of myeloma death, and only a small part of these differences was due to variation in competing risks, or ethnic or age distribution among the RHAs.

The overall myeloma-specific survival trends since 1990 are essentially the same as found in other studies: myeloma survival has increased significantly over time, most likely due to more effective therapies. However, we appear to have achieved higher survival times in more recent periods than some other studies. A Spanish study of myeloma patients [18] (median age 66 years) found that median survival increased from 24 months in 1990–1994, to 34 months in 2005–2009, and a Swedish study of patients diagnosed between 2008 and 2015 (median age 71) only found a 38% 5-year survival. In comparison, our median survival was almost the same in 1990–1994, but by 2005–2009 it had increased to about 5 years, and in 2010–2015 a 62% 5-year survival was achieved. This difference in survival in more recent times would be attenuated had the other studies used competing risks analysis: our survival times were usually slightly higher with competing risks analysis compared to Kaplan-Meier estimates. Although patients known to have died outside New Zealand (*n* = 19) and those with unbelievable ages or survival times (*n* = 43) were excluded from analysis, a small proportion of the recent difference may be due to incomplete exclusion of similar people.

When investigating survival by age at diagnosis using unadjusted Kaplan-Meier analysis, as was found in many other studies [13, 18–20], survival deteriorated with increasing age. Initially, Brenner et al. [21] and Ludwig et al. [22] showed improvements in survival in younger patients aged under 60 years or under 50 years respectively, but more recently, some improvements have been seen in older patients without consideration of competing risks [20, 23]. However, our multivariable competing risks analysis shows the trends in survival stratified by age are not so simple. In this study the greatest improvement in myeloma survival since 1990–1994 was seen in 60–69 year old patients, not in the youngest age group as has been previously reported [21, 22, 24]. In 1990–1994, our 80+ year old group had better myeloma survival than 60–79 year olds, but from 2000–2004 their survival was significantly poorer than other ages. The reason for this pattern of survival over time in the oldest age group is unclear, but over these years the frequency of competing events in people aged 80+ years decreased from about 40% in 1990–1999 to about 26% in 2005–2015. This could have been as a result of improvements in non-myeloma mortality in the elderly or because the coding of cause of death was more erratic in the 1990s in the elderly with multiple morbidities. Furthermore, any improvements in treatment over time that favored younger patients would also contribute to this trend.

It is difficult to compare our results for survival by ethnic group as many other studies have calculated relative survival without specifying the comparison population, or without using ethnic-specific life tables or their equivalent. One meta-analysis of clinical trial data [25] showed no significant difference in drug response by ethnic group (non-Hispanic white; Asian and Pacific Islanders; other) but the numbers of non-whites participating in the trials were very small. An English study of myeloma patients diagnosed from 2002 to 2008 showed that relative survival up to 5 years post diagnosis was slightly better in blacks and south Asians compared to whites [1]. The significantly increased risk of myeloma death in Māori using Cox proportional hazards regression was shown to be predominantly due to differences in competing risks among ethnic groups. The competing risks analysis found that the greatest improvement in myeloma survival since 1990–1994 occurred in Pacific Islanders and the least in NMNPI, and in 2010–2015 Māori did significantly better than the other ethnicities. Why this should have occurred is unknown as there is very limited evidence of biological differences in disease among ethnicities and there is no evidence of treatment efficacy varying with ethnicity [1].

A strength of this study is the compulsory notification of cancer to the NZCR since mid-1994. This has allowed the identification of a large, population-based cohort of myeloma patients covering all New Zealand without bias from referral centers and without the selection bias in clinical trials. The NZCR is considered almost complete but reliance on routine administrative data may also have other drawbacks. For example, although a lot of work has been done in New Zealand about the attribution and self-classification of ethnicity, it may still be misrecorded.

There are always caveats around the accuracy of death certificate reporting and with our mobile population some deaths overseas may not be recorded. Our mortality data include data, not only on main cause of death, but on contributing cancer and other contributory causes, and all these fields were searched for myeloma codes to determine death from myeloma. Obvious ‘immortals’ were excluded from analysis. That these exclusions were justified (and conservative) was verified by examination of the observed survival by age group from the Swedish Myeloma Registry [13].

## Conclusions

Improvements in myeloma survival have been unequal across subgroups and regions in New Zealand and it is essential to address health inequities to deliver tangible changes while still honoring individual healthcare preferences. Detailed information about utilization of chemotherapeutic agents and transplantation in New Zealand is not available. However, the obvious next step will be to examine how individual chemotherapy and stem cell transplantation utilization across subgroups defined by age, sex, ethnicity, geography and co-morbidity relate to survival and where improvements in myeloma management to address disparities might be made.

## Supplementary Information

**Additional file 1. Figure A1.** Kaplan-Meier curves for myeloma survival by year of diagnosis, age at diagnosis and ethnic group.

**Additional file 2. Table A1.** Multivariable Cox’s regression and CR* regression of myeloma survival for diagnosis age and year interaction.

## Data Availability

The datasets supporting the conclusions of this article are owned by the New Zealand Ministry of Health. Access to the raw data may be sought from the Statistical Services of the New Zealand Ministry of Health, by bona fide researchers with ethical and ministry approval.

## Abbreviations

NPPA: Named Patient Pharmaceutical Assessment
NZCR: New Zealand Cancer Registry
ICD: international classification of disease
NHI: national health index
PI: Pacific Islanders
NMNPI: non-Māori non-Pacific Islanders
RHA: regional health authority
HR: hazard ratio
SHR: subdistribution hazard ratio
