# Supplementary material for: Trends in survival from myeloma, 1990–2015: a competing risks analysis
Source: BMC Cancer. 2021 Jul 16;21:821. doi: 10.1186/s12885-021-08544-7 (PMC8283947; doi:10.1186/s12885-021-08544-7)
Supplement: Supplementary file 1 — Additional file 1. Figure A1. Kaplan-Meier curves for myeloma survival by year of diagnosis, age at diagnosis and ethnic group. [file 12885_2021_8544_MOESM1_ESM.doc]

Additional Figures

Crude survival after a myeloma diagnosis is shown by the Kaplan-Meier curves in Additional Figures 1-3. Survival has improved considerably since 1990-1994 (Figure A1) and survival worsens with increasing age at diagnosis (Figure A2). Survival appears better in Pacific Islanders than either Māori or Non Maori Non Pacific Island (NMNPI) peoples (Figure A3).

Figure A1. Kaplan-Meier curves for survival after a diagnosis of myeloma, by year of diagnosis.

Figure A2. Kaplan-Meier curves for survival after a diagnosis of myeloma, by age at diagnosis.

Figure A3. Kaplan-Meier curves for survival after a diagnosis of myeloma, by ethnic group.
